# Book Review

**DOI:** 10.1038/sj.bjc.6690788

**Published:** 1999-11

**Authors:** R I Freshney

**Affiliations:** CRC Department of Medical Oncology, University of Glasgow, Glasgow, UK


*British Journal of Cancer* (1999) **81**(5), 924
© 1999 Cancer Research Campaign
Article no. bjoc.1999.0788

### Book Review

### Human Cell Culture, Vol. I, Cancer Cell Lines, Part 1

Edited by JRW Masters and B Palsson Publication details, Kluwer Academic Publishers, Amsterdam, 1999

This book, made up of 16 invited chapters on specific types of cancer, is one of three parts of a volume on cancer cells. It has comprehensive tabular lists of cell lines from many sources, providing detail and insight that one would not get, or expect to get, from cell banks and databases, the only other collected resources of this nature. It contains a vast amount of background on pathology, molecular genetics and clinical history for an extensive list of cell lines. With other volumes in the series it will be a formidable resource for cell and molecular biology as well as for tissue culture technology.

It is a little difficult to comment on the completeness of a book such as this, as future parts will presumably cover the omissions of the current part. Apparently, there are two further parts of the cancer volume on the way, extending the list to some of the more common carcinomas, leukaemias and lymphomas; lung will be the subject of a third and separate part. It is a little surprising to find that Part 1 does not deal with the common cancers, which are dealt with in Part 2, but with other less common, though clearly equally important, types of cancer. A list detailing the contents or proposed contents of other books in the series, particularly within the volume on cancer, would have been useful, as the numbering in this series is a little difficult to follow. Volume I, listed at the back, and published in 1998, is simply called Human Cell Culture, while the current edition is called Volume I, Part 1. Surely the 1998 publication was Part 1? A simpler volume numbering system, whereby the current book would be Volume II, would have been preferable.

The chapters cover sarcomas, neuroblastomas, Ewing's sarcoma, mesothelioma, pancreatic, adrenal cortical, thyroid, pituitary, salivary, oesophageal, bladder, renal, skin (melanoma, three chapters, and non-melanoma, one chapter). I found these chapters easy to read, informative and well laid out, although not all address the questions on clinical relevance, accuracy of histopathology and significance of genetic abnormalities, proposed by the editors in the Introduction, and adhered to in the chapters on bladder cancer and renal cell cancer. This is not a technical manual of protocols, although some culture details are given. On the other hand, the tabular data are particularly valuable and provide a wealth of information on the source, pathology, culture characteristics and molecular genetics of the cell lines, not readily available elsewhere. It is a pity, however, that a few of the authors restrict their contributions to their own work, ignoring the cell lines existing elsewhere, unlike other authors in the book who have an admirably comprehensive approach.

A small quibble is that the references are all given in short form; I have to admit, although it takes more space, I prefer to see references written out in full so that one can assess, from the title, the relevance of the publication to one's own interests, and can identify research groups by authors, as the group head is often not the first author. It would also have been nice to see a few photographs, although some of these will already be in Hay's *Atlas of Human Cell Lines*.

All in all, this book will become a very valuable resource, assuming that successive books in the series fulfil the same needs for other tumours and normal cells. I commend the editors and authors for the amount of research and preparation that has gone into this very useful book and look forward eagerly to the continuing series.

*RI Freshney*
*CRC Department of Medical Oncology,*
*University of Glasgow,*
*Glasgow, UK*

